# Emotion Risk-Factor in Patients With Cardiac Diseases: The Role of Cognitive Emotion Regulation Strategies, Positive Affect and Negative Affect (A Case-Control Study)

**DOI:** 10.5539/gjhs.v8n1p173

**Published:** 2015-05-15

**Authors:** Mostafa Bahremand, Mostafa Alikhani, Ali Zakiei, Parisa Janjani, Abbas Aghaei

**Affiliations:** 1 School of Medicine, Kermanshah University of Medical Sciences, Kermanshah, Iran; 2 Substance Abuse Prevention Research Center, Kermanshah University of Medical Sciences, Kermanshah, Iran; 3 Social Developments and Health Promotion Research Center, Kermanshah University of Medical Sciences, Kermanshah, Iran; 4 Clinical Research Development Center, Imam Reza Hospital, Kermanshah University of Medical Sciences, Kermanshah, Iran

**Keywords:** cognitive emotion regulation, positive affect, negative affect, cardiac disease

## Abstract

Application of psychological interventions is essential in classic treatments for patient with cardiac diseases. The present study compared cognitive emotion regulation strategies, positive affect, and negative affect for cardiac patients with healthy subjects. This study was a case-control study. Fifty subjects were selected using convenient sampling method from cardiac (coronary artery disease) patients presenting in Imam Ali medical center of Kermanshah, Iran in the spring 2013. Fifty subjects accompanied the patients to the medical center, selected as control group, did not have any history of cardiac diseases. For collecting data, the cognitive emotion regulation questionnaire and positive and negative affect scales were used. For data analysis, multivariate analysis of variance (MANOVA) was applied using the SPSS statistical software (ver. 19.0). In all cognitive emotion regulation strategies, there was a significant difference between the two groups. A significant difference was also detected regarding positive affect between the two groups, but no significant difference was found regarding negative affect. We found as a result that, having poor emotion regulation strategies is a risk factor for developing heart diseases.

## 1. Introduction

Research evidence shows that the problematic regulation of the emotions (including anger and anxiety) has a major role in physical illnesses such as cardiovascular diseases (Begley, 1994). Many studies have been conducted on relationship between the effect of Type A personality and heart disease. The results have been inconsistent; therefore, some kind of meta-analysis was conducted to study the definite relationship of this personality type and cardiac diseases. The results showed that the type A behavior is not associated with cardiovascular disease (Low et al., 1998). According to these results, Denollet attempted to introduce other character types that can be associated with heart diseases; hence Denollet (1998) used the scl-90 questionnaire and the Marlowe Crown’s questionnaire of Social Acceptance on a number of cardiac patients. After the factor analysis, two subscales of negative emotions (anxiety, depression) and social inhibition were extracted. Denollet named this scale as the “D personality”. The social inhibition subscale implies the prohibition of emotional expressions in social relations and the avoidance of verbal and nonverbal communication with others. Individuals with high scores under social inhibition subscales might not be able to succeed in communicating with other people (Denollet, Pedersen, Vrints, & Conraads, 2006).

According to the model of Gross (2001), regulation of emotion includes all conscious and unconscious strategies that are used to increase, maintain or reduce the emotional, behavioral and cognitive components of an emotional response (J. J. Gross, 2002). It also refers to the ability to understand emotions, experience adjustment, and express emotions (J. J. Gross, 1998). Research has shown that emotion regulation problems are more prevalent in people with post-traumatic stress disorders (Tull et al., 2009). Training emotion regulation means as reducing and controlling positive and negative emotions of excitement. Results indicate that group training of emotion regulation has a positive impact on reducing self-damage, lack of emotion regulation and specific symptoms of borderline personality disorder as well as reducing symptoms of depression, anxiety and stress (Gratz & Gunderson, 2006). According to various studies, the emotion regulation causes an improvement in physical health in addition to its positive effects on emotional well-being (John & Gross, 2004; Jorgensen, Johnson, Kolodziej, & Schreer, 1996). In some studies it has been indicated that the problem of emotion regulation causes physical problems (J. Gross & O. John, 2003). Emotional inhibition is considered as a negative strategy in regulating emotions, leading to cardiovascular diseases (Butler et al., 2003) and the cognitive reevaluation of emotions as positive emotion regulation strategy leading to the reduction in blood pressure (Nyklíček & Vingerhoets, 2009).

Emotional experiences play a major role in various aspects of life such as coping with life changes and stressful events. Basically, emotional responses can be considered as biological reactions to the conditions which we consider as important or challenging opportunity, and these biological reactions are along with the response we give to those environmental events (Nadia Garnefski et al., 2002).

Although emotions have biological basis, individuals are able to affect the ways which these emotions are expressed. These abilities are called emotion regulation and include internal and external processes which are responsible for an individual’s controlling, evaluating and changing emotional reactions in the realization of his goals (Thompson, 1994). Therefore, emotion regulation is considered as a fundamental principle in starting, evaluating and organizing the adaptive behavior and also preventing negative emotions and maladaptive behaviors (Cicchetti, Ackerman, & Izard, 1995). Review of psychological literature indicates that emotion regulation is an important factor in determining one’s health and having a successful performance in social interaction (Thompson, 1994; Cicchetti et al., 1995) so that the ineffective regulatory traits of emotional experiences is evident in all psychological disorders. For example, the schizophrenia disorder, involves extreme and uncontrollable emotion; neuroticism is dependent on anxiety; anti-society, is rooted in the lack of overt emotion and even mental retardation is considered as emotional retardation (J. J. Gross, 1998). Furthermore, the recent reviews indicate that emotional malfunctioning occurs in all disorders of axis I and most of the axis II disorders (Aldao, Nolen-Hoeksema, & Schweizer, 2010). Structure of emotion regulation is a complex concept that includes a wide range of biological, social, behavioral, and cognitive processes as well as alert and unconscious cognitive processes (N. Garnefski, Kraaij, & Spinhoven, 2001; J. J. Gross, 2002) and refers to their comprehension ability to understand, modification and expressing emotional experiences (J. J. Gross, 1998) or referring to their values being diminished, sustained, or elevated (Jermann, 2006; J. J. Gross, 1999). Any difficulty or deficiency in emotion regulation can make an individual vulnerable to mental disorders such as depression and anxiety (Nadia Garnefski, Boon, & Kraaij, 2003). Thus, it can be concluded that emotion regulation is a key determinant of mental well-being, effective functioning, adapting with stressful life events, and quality of life. Emotion regulation, as one of the psychological factors, has been taken into account by many researchers (Mayer, 1997).

According to the studies, one of the factors affecting the increase in heart disease are emotions such as anger, and negative emotions like hostility, the presence of these emotions and excitement, even in cardiac patients exacerbates the disease, so we can help preventing or treat cardiac patients by identifying positive and negative emotions, and examining the role of emotion regulation. However, the results are inconsistent in this case, some studies didn’t find any relationship between the factors such as hostility, anxiety, and competing, therefore, it is important to conduct new researches in this field and this research can be useful in this regard. Therefore, it seems that the detection and diagnosis of emotions and the way of regulating emotions in cardiac patients is of great importance, thus, the present study sought to answer the questions that in what level are the positive affect and negative affect in cardiac patients and how different are they in non-patients? And how the emotion regulation differs in patients compared to non-patients?

## 2. Materials and Methods

This study was a case-control study. The study had two populations, the first consisted of all patients with a cardiac disease (of a coronary artery disease type) who presented to Imam Ali medical center in Kermanshah in spring 2013in Iran and had previously been diagnosed with heart disease by cardiologist. The selected individuals were diagnosed with heart disease on their first visit to the center. Others with prior visitations or those with more than one visit were excluded from the study. The second population consisted of all the individuals who accompanied the patients visiting the medical center and according to the doctor, are not afflicted with any certain chronic diseases such as heart disease. The two groups were matched in terms of genetics, age and gender. In some cases, due to the heterogeneity of the patient and the accompany, a family member or a patients’ relative who was a better match in terms of age and sex, was invited to fill out the questionnaire of the study. To conduct the study, two samples of 50 individuals were selected using convenient sampling method. As a result of submission of incomplete questionnaire, an additional 20 individuals were also considered which brought up the total size of the sample to 120 individuals. At the end, the analysis was performed on 100 individuals. The population consisted of 55 females and 45 males with average age of 38.27 years and standard deviation of 13.15 years. The questionnaires were distributed after applying the necessary adjustments and final selection of the participants. Then the necessary descriptions on how to fill the questionnaires were presented to the participants. The participants were told to ask for further explanation from the researcher if they confronted a problem. Upon declaration of consent in participating in this research, and receiving the necessary assurances in confidentiality of the content of replies, the selected individuals started to fill out the questionnaires. The questionnaire was read out loud to uneducated individuals and the forms were filled out by the researchers. After completing the questionnaire by the subjects which was set individually and in the presence of the researcher, the questionnaires were collected.

### 2.1 Research Tool

#### 2.1.1 Positive and Negative Affect Scale (PANAS-X)

The long form is a list of positive and negative affect (PANAS-X, Watson and Clark, 1994) composed of 60-item self-report test that measures 4 major scales of negative emotions (fear, hostility, guilt, and sadness), 3 major scales of positive emotions (happiness, self-confidence, and precision), and 4 other emotional states (shyness, fatigue, calmness, and surprise), in addition to measuring the two major factors of positive and negative affect. The items are single words that are scored by the subject based on his/her reports his/her own experience on a five-point scale (1 = not at all; 2 = low; 3 = moderate; 4 = much; 5 = very much). The creators of the test use 8 different time orders: now, today, during the last few days, a few weeks ago, last month, last year, and generally to assess the state affect and attributive affect. For the compliance of the test with the trait or state form the guidelines should be modified on the state related manner (how do you usually feel?) or trait-related manner like (how do you feel today?). That is, the respondents are asked to recall their affect either generally or in specific intervals. This questionnaire was composed in regards to the aims of the study given the instructions (this list includes some of the words that describe different feelings and emotions. Read each set of words and identify that over the past few weeks, how much you have had these feelings and emotions). In a research by Mohammadi et al, which aimed on the translation and Farsi standardization of the questionnaire, the internal consistency coefficients of (Cronbach’s alpha) were respectively obtained for the instruction “now” between 0.72 to 0.93, with the instruction “last week” between 0.74 and 0.90 and the instruction “last year” between 0.74 and 0.90, respectively (Mohammadi, 2012).

#### 2.1.2 Cognitive Emotion Regulation Questionnaire (CERQ)

CERQ is a 36-item questionnaire which was prepared by Garenfesky et al. The questionnaire is used to identify the cognitive emotion regulation of individuals (or cognitive strategies) after experiencing negative events and situations. Unlike other questionnaires that do not differentiate between the individual’s acts and thoughts, this questionnaire assesses what people tend to think about during and after experiencing threatening events. The Cognitive Emotion Regulation Questionnaire contains 9 distinct subscales that some of the cognitive emotion regulation strategies and cognitive strategies, some of which are negative or non-adaptive such as self-blame, rumination, catastrophizing, and other-blame. Other strategies are positive or adaptive that include acceptance, positive refocusing, refocus on planning, positive reappraisal, and putting into perspective. Each subscale consists of four elements, which range from 1 (almost never) to 5 (almost always). The higher the score of a subscale, the intended cognitive strategy is used more (Markarian, Pickett, Deveson, & Kanona, 2013). To assess the reliability of this questionnaire Garenfesky et al. calculated internal consistency of the 9 subscales of the questionnaire using Cronbach’s alpha coefficients. The Cronbach’s alpha coefficient for the subscale of self-blame was 0.81, acceptance 0.80, rumination 0.83, positive thinking load 0.81, refocusing on planning 0.81, refocus on planning 0.72, putting into perspective 0.79, catastrophizing 0.71 and other-blame 0.68, which all indicated a good reliability of the questionnaire. They also calculated the test-retest reliability of the subscales in a 14-month period. Results indicated that test-retest correlations range between 0.48 (refocus on planning) and 0.65 (other-blame). This questionnaire has been standardized by Samani and Sadeghi (2011).

The multivariate analysis of variance test was applied to analyze data using statistical software SPSS-19.

## 3. Findings

As shown in Table 1, the mean values for strategies of positive refocusing, positive reappraisal, rumination and acceptance are higher in healthy subjects than the cardiac patients. Mean values for strategies of other-blaming, self-blaming and catastrophizing are higher in cardiac patients compared to healthy subjects. Also, in the positive affect, the mean value is higher for the healthy individuals compared to the cardiac patients. The results also indicate that in examining each of the variables between the two groups, the t-test was used so that the results showed that in all cognitive emotion regulation strategies, there is significant difference between the two groups of cardiac patients and healthy individuals, the results also indicated that the mean difference between the two groups is significant in positive affect but in negative affect there is no significant difference between the two groups.

**Table 1:** Mean and standard deviation of cognitive emotion regulation strategies, positive affect, and negative affect and results of the multivariate analysis of variance (MANOVA) to compare the two groups of cardiac patients and healthy individuals

Variable		Cardiac patients	Healthy subjects	t	p-value

		M (SD)	M (SD)		
**Cognitive Emotion Regulation Strategies**	Positive refocusing	27.64 (6.24)	37.78 (6.61)	7.88	0.001
	Positive reappraisal	16.34 (3.14)	20.38 (4.63)	5.1	0.001
	Other-blame	15.64 (3.11)	11.6 (3.98)	5.64	0.001
	Self-blame	9.82 (2.76)	6.26 (3.03)	6.13	0.001
	Rumination	12.66 (2.68)	16.2 (3.44)	5.73	0.001
	Catastrophizing	12.24 (3.16)	10.38 (2.94)	3.04	0.003
	Acceptance	9.7 (2.56)	11.78 (3.45)	3.41	0.001
**Positive Affect**		67.28 (19.72)	78.26 (17.92)	2.91	0.004
**Negative Affect**		91.68 (21.77)	88.9 (23.11)	0.62	0.537

## 4. Discussion

Annually, about 20 million people worldwide survive a heart attack, and the majority of them need expensive care. The cost of heart surgeries and also rehabilitation costs are on the rise daily. The disease occurs when people are at the age of economical productivity and maximum efficiency, and on the other hand, thousands of days lost as a result of absences from work due to coronary artery disease, may also cause damage to work and production, thereby causing an increase in the economic burden of illness to the society. Given that the psychology services, counseling, and cognitive therapies are as the general principles for rehabilitating coronary artery patients, the use of psychological treatments along with medical and surgical treatments of cardiac patients seems essential. Studies show that the phenomenon of cognitive mechanisms leads to emotional disorders and subsequent cardiovascular or heart attacks or even sudden death. The development of a state of anger, stress, and fear are the causes of intense and sometimes uncontrollable excitements. So far, several researches have been done on the impact of negative emotions such as; fear, depression, anger, and hostility on heart. The present study, therefore, was conducted for the purpose of comparing the cognitive emotion regulation strategies, positive and negative effects in cardiac patients and healthy individuals. The results indicated that positive refocusing strategies, positive reappraisal, rumination and acceptance was higher in the healthy group than the cardiac patients, and in strategies of blaming others, self-blame, and catastrophizing, the mean value is higher in cardiac patients than the healthy individuals, and in the in positive affect, the number of healthy individuals are more than the cardiac patients. The results indicated that there is no significant difference in the negative affect of cardiac patients and healthy individuals.

So far, no other research has been conducted on this topic, but some studies like the research done by Gharakhani, and But the results of this study also indicated that there is no significant relationship between the type A personality and risk of coronary artery disease. In a research conducted by Day et al (2005), the results indicated that in patients with depression and anxiety, their negative emotions are effective in the increased incidences of heart disease, and according to the patients, their mental state is effective in the formation of heart disease (Day, Freedland, & Carney, 2005). In fact, we can say that the results are inconsistent with the results of previous researches.

Gross in his theory showed that the individual differences in using different styles of cognitive emotion regulation will carry out different emotional, cognitive, and social consequences. For example, the use of reappraisal styles is related to positive emotional experiences and better intrapersonal practices, and higher well-being (J. Gross & O. John, 2003). Overall, the research literature show that individuals who use poor cognitive strategies such rumination, catastrophizing and self-blame, are more vulnerable to emotional problems, than any other people, while those with optimal strategies, such as positive reappraisal, are less vulnerable (Nadia Garnefski & Kraaij, 2006), and self-blame strategies, catastrophizing, and positive reappraisal are among the strong predictors of negative emotional experiences (Martin & Dahlen, 2005). Thus, the present findings support the assumption that people with heart disease, use inconsistent strategies to deal with life problems and negative events and the vulnerability of aspects of pathology is high in them, it can be said that these strategies are adverse risk factors for cardiovascular disease.

Cognitive emotion regulation strategies appear to be a critical factor in individual’s health. It is believed that cognitive emotion regulation strategies helps people to regulate arousal and negative emotions (J. Gross & O. John, 2003; V. Kraaij, Pruymboom, & Garnefski, 2002; Campbell-Sills & Barlow, 2007) and optimal utilization of cognitive adapted discipline strategies such as the of reappraisal causes a decrease in negative emotions and an increase in positive feelings and adaptive behavior (Rottenberg & Gross, 2003). In general, a number of research findings indicate a strong relationship between cognitive emotion regulation with emotional behavioral disorders (Rottenberg & Gross, 2003; Bargh & Williams, n.d.) and psychopathology (N. Garnefski et al., 2001; J. Gross & O. John, 2003; V. Kraaij et al., 2010; Nadia Garnefski, Teerds, Kraaij, Legerstee, & van den Kommer, 2004). Furthermore, the research results show that effective emotion regulation has desirable outcomes in mental health, psychological well-being, physical health and interpersonal relationships (Hasani, 2012).

The results of the study showed that positive affect on heart patients are less than non-patients, studies have shown that positive affect through strengthening the immune system plays a role in improving health. Positive affect includes the tendency to conflict and confrontation with the environment, including social environment. People live up to life with high positive affect, actively, strongly, with passion, joy, and confidence, seeking companionship with others and enjoy it, and have trust and satisfaction in their social interactions. These people like dramatic experiences. On the other hand, people with low positive affect, have lack of energy, enthusiasm, and confidence. They are introvert and unsociable, avoid passionate experiences and are totally doubtful about active engagement with the environment. However, the research results show that there is no difference in negative affect between patients and healthy individuals. Some other studies, have reached similar finding (Low et al., 1998), and as it was stated before; the results obtained by the researches in this area are inconsistent.

According to the results of this research, we can say that having poor emotion regulation strategies is a risk factor for succumbing to cardiovascular diseases; therefore, it is suggested to help the health of the population by the recognition of emotion regulation strategies in people and rectifying them in this context. In generalizing the results we should be cautious, because this study was conducted among patients who have referred for treatment of their disease, therefore, future research should be done on the broader community.
